# Ubiquitin specific protease 38 aggravates pathological cardiac remodeling by stabilizing phospho-TBK1

**DOI:** 10.7150/ijbs.85562

**Published:** 2024-02-25

**Authors:** Zheng Xiao, Chang Dai, Tingting Yu, Jun Zhu, Yucheng Pan, Wei Shuai, Bin Kong, He Huang

**Affiliations:** 1 Department of Cardiology, Renmin Hospital of Wuhan University, Wuhan, China.; 2 Hubei Key Laboratory of Cardiology, Wuhan, China.; 3 Cardiovascular Research Institute of Wuhan University, Wuhan, China.; 4 Department of Respiratory Medicine, Hubei RongJun Hospital, Wuhan, China.

**Keywords:** Pathological cardiac remodeling, Heart failure, Ubiquitin specific protease 38, TANK-binding kinase 1

## Abstract

Chronic pressure overload can cause pathological cardiac remodeling and eventually heart failure. The ubiquitin specific protease (USP) family proteins play a prominent role in regulating substrate protein degradation and cardiac structural and functional homeostasis. Although USP38 is expressed in the heart, uncertainty exists regarding the function of USP38 in pathological cardiac remodeling. We constructed and generated cardiac specific USP38 knockout mice and cardiac specific USP38 overexpression mice to assess the role of USP38 in pathological cardiac remodeling. Furthermore, we used co-immunoprecipitation (Co-IP) assays and western blot analysis to identify the molecular interaction events. Here, we reported that the expression of USP38 is significantly elevated under a hypertrophic condition in vivo and in vitro. USP38 deletion significantly mitigates cardiomyocyte enlargement in vitro and hypertrophic effect induced by pressure overload, while overexpression of USP38 markedly aggravates cardiac hypertrophy and remodeling. Mechanistically, USP38 interacts with TANK-binding kinase 1 (TBK1) and removes K48-linked polyubiquitination of TBK1, stabilizing p-TBK1 and promoting the activation of its downstream mediators. Overexpression of TBK1 in the heart of cardiac specific USP38 knockout mice partially counteracts the benefit of USP38 deletion on pathological cardiac remodeling. The TBK1 inhibitor Amlexanox significantly alleviates pressure overload induced-cardiac hypertrophy and myocardial fibrosis in mice with USP38 overexpression. Our results demonstrate that USP38 serves as a positive regulator of pathological cardiac remodeling and suggest that targeting the USP38-TBK1 axis is a promising treatment strategy for hypertrophic heart failure.

## 1. Introduction

Heart failure, characterized by the inability of the heart to pump sufficient blood and/or congestion, is a complex clinical syndrome at the end of the pathological process of various cardiovascular diseases and has become a major health burden worldwide^1^. It is well known that pathological cardiac hypertrophy is pivotal factor of heart failure, usually elicited by various stimuli such as neuro-hormones and pressure overload^2^, ^3^. Notably, there is increasing evidences that Tank-binding kinase 1 (TBK1) plays an important role in pathological cardiac remodeling^4^, ^5^. TBK1 belongs to the non-canonical IκB kinases family, and it has the powerful competence to activate multiple signaling cascades, including the Akt (protein kinase B)-, MAPK (mitogen activated protein kinase)-, NF-κB (nuclear factor kappa B)-, IFN (interferons)- and TNF (tumor necrosis factor)-signaling pathways, regulating oncogenesis, neurodegenerative diseases, metabolic diseases and cardiovascular diseases^6^, ^7^. Especially, Akt is a serine/threonine protein kinase, a key signaling transduction that regulates proliferation, migration, cell growth and metabolism^8^. Akt is activated by phosphorylation on Ser473 and it phosphorylates a variety of downstream signaling protein including Glycogen synthase-3β (GSK3β) and mammalian target of rapamycin (mTOR)^9^. GSK3β is expressed in numerous tissues and its phosphorylation on Ser9 inactivated GSK3 by blocking the active site^10^. mTOR belongs to the phosphoinositide kinase-related kinase (PIKK) family, and is phosphorylated at Ser2448 by the Akt signaling^11^. The Akt-GSK3β/mTOR signaling pathway is deeply involved in the progress of cardiac hypertrophy and remodeling. Under pressure overload stimulation, the phosphorylation level of Akt and its downstream molecule, such as GSK3β and mTOR, were upregulated^4^, ^12^. Furthermore, activation and function of TBK1 are regulated by post-translational modifications (PTMs) such as ubiquitination, phosphorylation, hydroxylation and acetylation^13^-^15^. Recently, an accumulating study have reported that the degradation of TBK1 is closely related to its K48-linked polyubiquitination^16^, ^17^. Thus, attention should be paid to the ubiquitination mechanism of TBK1 in pathological cardiac remodeling.

Ubiquitin specific proteases (USPs) are the main members of the deubiquitinating enzymes (DUBs) that can count-regulate the process of ubiquitination by cleaving ubiquitin from substrate proteins^18^. USPs play prominent roles in antivirus immunity, inflammation, metabolic disorders, neurological diseases and cancer^19^-^21^. Recently, accumulating evidence have confirmed the function of USP4, USP10, USP18, and USP25 in the regulation of pathological cardiac hypertrophy and remodeling^22^-^25^, suggesting a possible involvement of the USP family in pathological cardiac remodeling. Among the USP members, USP38 plays a crucial role in various diseases. For example, USP38 deubiquitinates JunB protein, which contributes to the development of allergic asthma. Knockout of USP38 in mice increased resistance to asthma^26^. In addition, USP38 has been shown to promote cell proliferation by stabilizing LSD1 or c-Myc proteins^27^, ^28^. However, it remains unknown whether USP38 is associated with pathological cardiac remodeling.

Here, we used animal and cell models of hypertrophic growth to explore the function of USP38 in pressure overload-induced cardiac remodeling and its impact on primary neonatal rat cardiomyocytes (NRCMs) treated with angiotensin II (Ang II). Our findings revealed that USP38 deficiency improves cardiac dysfunction and pathological cardiac remodeling caused by aortic banding (AB). Conversely, overexpression of USP38 accelerates the progression of cardiac hypertrophy and myocardial fibrosis. Mechanistically, USP38 prevents the proteasomal degradation of p-TBK1, thus activating the downstream Akt-GSK3β/mTOR signaling pathways. Together, we unveiled a novel role of USP38 in pressure overload induced cardiac remodeling and its potential as a therapeutic target for pathological cardiac remodeling and heart failure.

## Methods

### Animals and animal model

All experimental animal procedures in our study were performed in accordance with the institutional ethical guidelines and approved by the Animal Experimental Ethical Committee of Renmin Hospital of Wuhan University (Protocol No. WDRM20221207B). To establish a pressure overload-induced cardiac hypertrophic model, aortic banding (AB) surgery was performed according to previously described methods^29^. Briefly, male mice (8- to 10-week-old) were anesthetized intraperitoneally with 40 mg/kg pentobarbital. After losing the toe pinch reflex, a 27G needle was used to ligate the thoracic aorta. The sham surgery was performed in the same procedure except for aortic constriction. Mice were fed for another 4 weeks after this operation. All mice were housed under a temperature of 22 ± 2 °C and a standard 12 hours dark/light cycles with free access to food and water ad libitum.

### Generation of cardiac specific USP38 knockout (USP38^cko^) mice

The USP38^cko^ mouse construction was performed at Cyagen Biotechnology. Briefly, the in vitro synthesized sgRNA, the donor vector containing loxP sites and Cas9 mRNA were co-injected into mouse zygotes, which were then transferred to pseudopregnant mice. The genotypic identification was carried out with the following primers: USP38 F: 5'-ATGATCGGAGGTTTCCTTGTGTTG-3' and USP38 R: 5'-TCTGATGTCTGAGTATCAACGAAGA-3'. The genotype of USP38^cko^ was maintained by crossing the USP38^fl/fl^ mouse and the α-MHC-Cre tool mouse. Finally, the USP38^cko^ and USP38^fl/fl^ mice were used in subsequent studies.

### Generation of cardiac specific USP38 transgenic (USP38-TG) mice

The sgRNA to mouse ROSA26 gene, the vector containing “Kozak-Mouse USP38 CDS” sequence and Cas9 mRNA were injected into mouse eggs to generate targeted conditional knockin offspring. The mouse was identified using the following primers sequence: USP38 F4: 5'-ATCTGCTTCCTGTTCGTTCCGAC-3' and R4: 5'-CTTTATTAGCCAGAAGTCAGATGC-3'. The mouse was bred to cross with α-MHC-Cre tool mouse to maintain the genotype of USP38 overexpression.

### Recombinant adeno-associated virus (rAAV9)-Tbk1 overexpression mice

The Tbk1 and GFP genes were amplified from cDNA and ligated into the vector (cTnT-3Flag-T2A-EGFP), and constructed plasmid was packaged into recombinant adeno-associated virus serotype 9 (rAAV9) vector carrying cardiac troponin T (cTnT) promotors to drive the expression of GFP and TBK1. According to previous study, viral solution (5×10^11^ vg/ml, 200μl/mouse) was slowly injected via tail vein 1 week after surgery^30^.

### TBK1 inhibitor (Amlexanox) treatment

After AB surgery of 2 weeks, Amlexanox (MedChemExpress, HY-B0713, Shanghai, China), a specific TBK1 inhibitor, was dissolved in DMSO, and then intraperitoneally injected into USP38-TG mice at previous reported dose (25 mg/kg)^31^. The control group was administrated with the corresponding dose of DMSO according to body weight.

### Cell culture

Primary neonatal rat cardiomyocytes (NRCMs) were prepared from Sprague Dawley rats born within three days according to previously described methods^25^. NRCMs were cultured in medium containing 10 % fetal bovine serum (10099, Gibco, Australia), 5-bromodeoxyuridine (0.1mM) and 1 % penicillin-streptomycin solution (SV30010, Hyclone, USA). The medium was replaced for serum-free before treatment with Ang II (1 μM). In order to knockdown or overexpress specific target genes, recombinant AdUSP38 vector were generated by introducing rat USP38 cDNA into a replication-defective adenoviral vector, and AdshUSP38 adenovirus was prepared by rat harpin USP38. AdGFP and AdshRNA were used as control, and the efficiency of these adenoviruses were validated. NRCMs were transfected by AdshUSP38 or AdshRNA at a multiplicity of infection (MOI) of 40 for 24 hours, and AdUSP38 or AdGFP transfected NRCMs with 20 MOI. In addition, the plasmids, encoding Myc, Myc-USP38 and Myc-USP38 (H857A) mutant, were constructed and transfected NRCMs using NEOFECT DNA transfection reagent (TF20121201, Beijing, China).

### Echocardiography

The cardiac function of mice was evaluated at the 4 weeks after surgery with echocardiography (Vinno Technology, Suzhou, China) equipped with a 23-MHz line array transducer. After being under anesthesia (1.5 isoflurane/100 oxygen), mice were placed in supine position on a 37 °C heating pad. These echocardiography parameters such as left ventricular internal diameter at end-systole (LVIDs), left ventricular internal diameter at end-diastole (LVIDd), ejection fraction (EF) and fractional shorting (FS) were collected from three to five cardiac cycle.

### Histologic analysis

Prior to collection tissues, mice were anaesthetized with 1.5 % isoflurane and then sacrificed by cervical dislocation. The body weight (BW), heart weight (HW), lung weight (LW) and tibia length (TL) were measured to evaluate HW/BW, HW/TL and LW/BW ratio. Subsequently, the hearts were fixed in 4 % paraformaldehyde, and made into 5 μm thick section. According to the standard procedure, the hematoxylin-eosin (H&E) staining, Wheat germ agglutinin (WGA) and Masson's staining were performed to evaluate the cardiomyocyte cross-sectional area and myocardial fibrosis, which were calculated using Image J.

### Immunofluorescence staining

Immunofluorescence staining was performed on NRCMs grown on coverslips. NRCMs were fixed with 4 % paraformaldehyde for 15 min and blocked with 3 % BSA in PBS for 30 min at room temperature. For α-actinin staining, NRCMs were incubated at 4 °C for overnight with a rabbit anti-α-actinin antibody (GB111556, Servicebio, Wuhan, China) and a corresponding anti-rabbit Cy3 antibody (GB21302, Servicebio, Wuhan, China) for 1 hour at room temperature. The image was obtained via the fluorescence microscope. The surface area of NRCMs were depicted using Image J. For co-localization of USP38 and TBK1, NRCMs were incubated with rabbit anti-USP38 at 4 °C for overnight, then incubated with corresponding anti-rabbit HRP-labled antibody for 1 hour, followed by Cy3-Tyramide (GB1223, Servicebio, Wuhan, China) for 1 hour at room temperature. Subsequently, NRCMs were incubated with rabbit anti-TBK1 antibody at 4 °C for overnight, then incubated with anti-rabbit Alexa Fluro 488 antibody (GB25303, Servicebio, Wuhan, China) for 1 hour at room temperature. The nucleus was stained with DAPI (GB1012, Servicebio, Wuhan, China) for 10 min. The image was obtained via the confocal scanning microscope (NIKON Eclipse TI, Tokyo, Japan).

### Immunoprecipitation (IP) assays

Co-immunoprecipitation (Co-IP) was performed according to previous report^32^. Briefly, NRCMs were lysed in lysis buffer with PMSF and Cocktail on ice for 30 min. After concentration, the target protein was precipitated by the corresponding antibody and magnetic protein A/G (L-1004, Biolinkedin, Shanghai, China). Finally, the immune complex was analyzed by western blot using the indicated primary antibodies and secondary antibodies. The same procedure was performed using mouse heart tissues.

### Ubiquitination assay

To evaluate the endogenous ubiquitination level of TBK1, NRCMs were transduced with AdUSP38, AdGFP, AdshUSP38 and AdshRNA for 24 hours, respectively. After Ang II or PBS stimulation for 48 hours, the cell lysates were harvested. MG132 (MedChemExpress, HY-13259, Shanghai, China) was used 6 hours before NRCMs being lysed. The cell lysates were incubated with anti-TBK1 antibody for overnight, and the incubated with magnetic protein A/G beads for 2 hours. Similarly, the lysates of mouse heart tissue were obtained. The ubiquitination level was detected using ubiquitin antibody.

### Quantitative Real-time PCR (qPCR)

Total RNA was extracted from mouse heart samples using RNAiso Plus reagent (9190, Takara, Japan). Subsequently, RNA was reverse-transcribed into cDNA with the SweScript All-in-One-First-Strand RT SuperMix (G3370, Servicebio, Wuhan, China). Then, qPCR was conducted with 2X Universal Bule SYBR Green qPCR Master Mix (G3326, Servicebio, Wuhan, China) on Real-Time PCR System (Applied Biosystems ViiA 7 Dx, Waltham, MA). GAPDH was used as internal reference. Primer sequences were listed in **Supplementary Table S1**.

### Western blot analysis

Total proteins were extracted from mouse heart tissues or cells. Protein concentrations were determined by BCA methods. Subsequently, the protein extracts were separated by 8-12 % SDS-PAGE and transferred onto PVDF members. After soaked in 5 % skim milk for 2 hours, the members were immunoblotted with indicated primary antibodies for overnight **(Supplementary Table S2).** The next day, the members were incubated with the secondary antibody for 1 hour. Finally, the protein signals of the members were visualized by enhanced chemiluminescence (BL523, Biosharp, China). Each protein was normalized by GAPDH.

### Microarray data

The analysis of microarray data (GSE3586) was collected from GEO database, and the different expression genes (DEGs) was acquired via GEO2R. The top 18 DEGs of ubiquitin-specific proteases were selected to make heat map.

### STRING and Cytoscape integrative network analysis

The STRING database (cn.string-db.org) was utilized to established a protein-protein interaction involving USP38, TBK1 and Akt-GSK3β/mTOR, and the results were visualized using Cytoscape (cytoscape.org).

### Structure-based protein interaction interface analysis between USP38 and TBK1

Protein sequences for USP38 (4RXX, Chain A) and TBK1 (4IM0, Chain A) were obtained from the Protein Data Bank (www.rcsb.org). The SWISS-MODEL tool (swissmodel.expasy.org) was utilized to predict homology structure models based on protein sequence. The protein sequences were submitted to the Z-DOCK (zdock.umassmed.edu) in order to predict their potential interaction interfaces. The prediction results were visualized using PDBBePISA (www.ebi.ac.uk).

### Statistics

All data were displayed as mean ± standard deviation (SD) and analyzed using GraphPad Prism 9.0.0 software. For data with a gaussian distribution, two sets of data used unpaired, two-tailed Student's *t*-test. Multiple group comparisons were performed by a one-way analysis of variance followed by Tukey's multiple comparisons test. *P*<0.05 was considered statistically significant.

## Results

### The expression of USP38 is upregulated in human failing hearts, murine hypertrophic hearts and Ang II exposed cardiomyocytes

**Figure 1:**
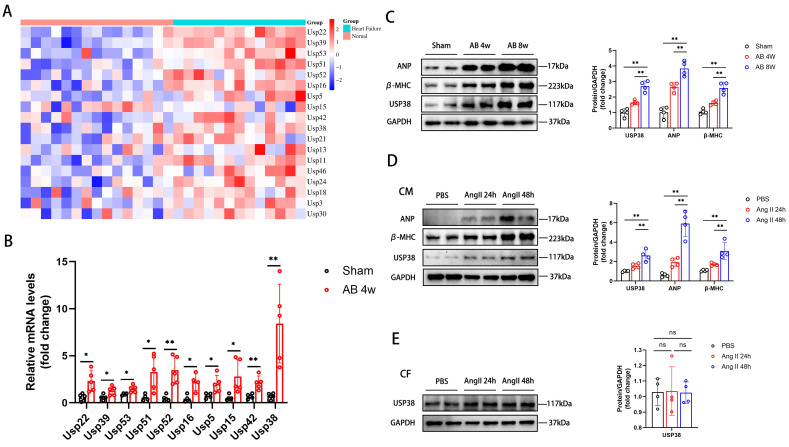
** USP38 expression is increased in murine hypertrophic hearts and Ang II exposed hypertrophic myocardium.** (A) The heatmap upregulation gene expression profile of ubiquitin-specific proteases (USPs) in heart tissues of human heart failure. (B) The mRNA expression of top ten USPs in pressure overload induced mouse heart tissues (n=4-5). (C) Western blot bands and statistical analysis of ANP, β-MHC and USP38 in sham-operated mice and aortic banding (AB) mice heart tissues (n=4). (D) Western blot bands and statistical analysis of ANP, β-MHC and USP38 in neonatal rat cardiomyocytes (NRCMs) exposed to phosphate buffered saline (PBS) or angiotensin II (Ang II) for the indicated time (n=4). (E) Western blot bands and statistical analysis of USP38 in neonatal rat cardiac fibroblasts (NRCFs) exposed to PBS or Ang II for the indicated time (n=4). Data was calculated by one-way analysis of variance (Tukey's multiple comparisons test) or Student's *t*-test (unpaired, two-tailed, two groups). *P < 0.05, **P < 0.01.

Firstly, we downloaded GSE3586 from the GEO database to explore the function of ubiquitin-specific proteases (USPs) in heart failure. A series of USPs were upregulated in patients with heart failure **(Figure 1A)**. Among the top ten upregulated genes, USP38 mRNA levels were most significantly elevated in the heart of mouse induced by pressure overload **(Figure 1B)**. Secondly, western blot analysis showed a significant increase in USP38 protein levels in pressure overload-induced hypertrophic model compared to the sham group **(Figure 1C)**. After Ang II stimulation, the protein levels of ANP, β-MHC and USP38 in neonatal rat cardiomyocytes (NRCMs) were markedly increased, while no such phenomenon was observed in the USP38 protein expression of neonatal rat cardiac fibroblasts (CFs) **(Figure 1D and 1E).** These results indicate that the upregulation of USP38 may be associated with the pathogenesis of cardiac hypertrophy.

### Effect of USP38 in Ang II-induced hypertrophic cardiomyocytes

**Figure 2:**
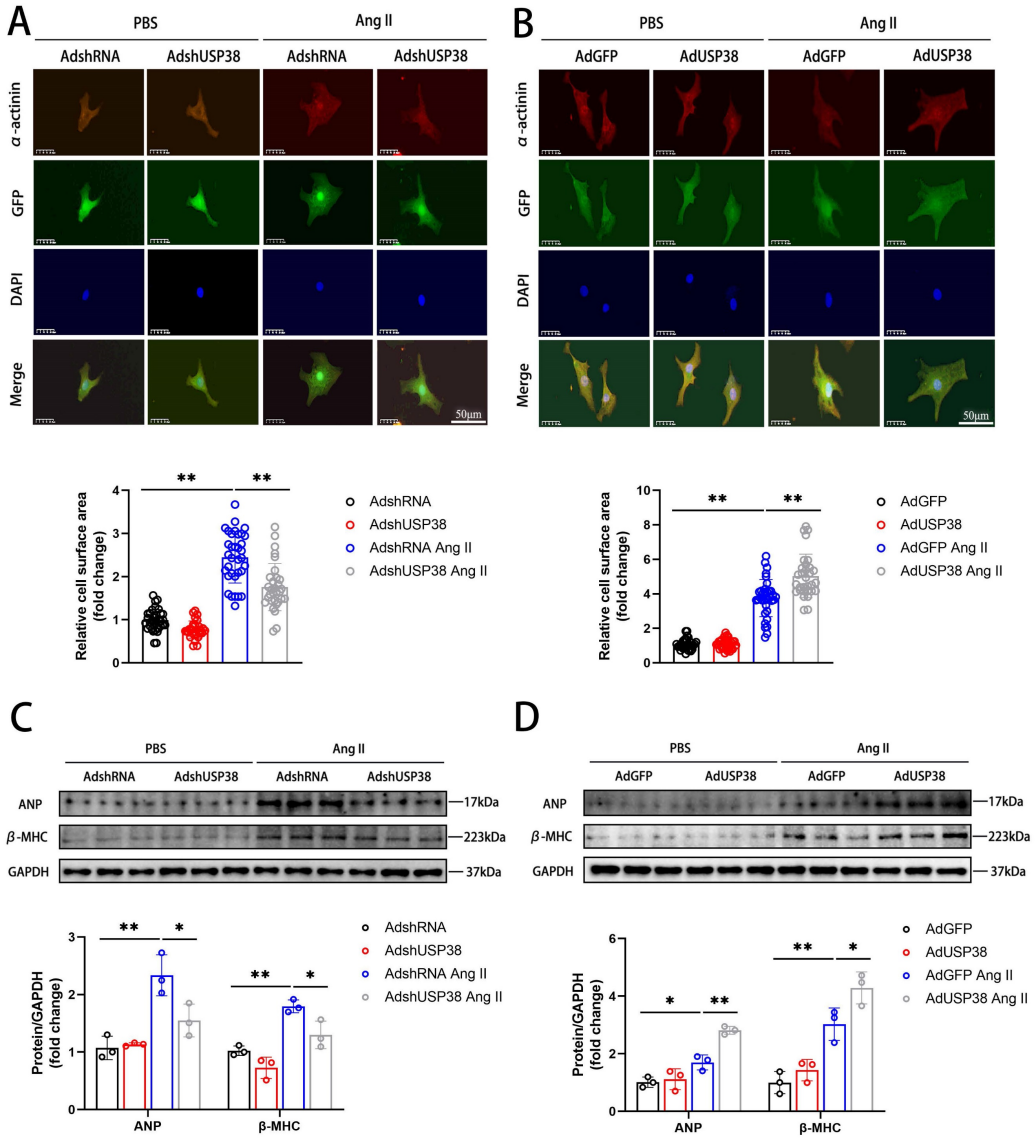
** USP38 aggravates Ang II-induced cardiomyocyte hypertrophy in vitro.** (A) Representative immunofluorescence images and statistical analysis of α-actinin stating of NRCMs which transfected with AdshRNA or AdshUSP38 (adenovirus expressing short hairpin RNA targeting USP38) and treated with PBS or Ang II (n≥30). Scale bar, 50 μm. (B) Representative immunofluorescence images and statistical analysis of α-actinin stating of NRCMs which transfected with AdGFP (adenovirus expressing green fluorescent protein) or AdUSP38 (adenovirus expressing USP38) and treated with PBS or Ang II (n≥30). Scale bar, 50 μm. (C) Western blot bands and statistical analysis of ANP and β-MHC proteins of NRCMs which transfected with AdshRNA or AdshUSP38 and treated with PBS or Ang II (n=3). (D) Western blot bands and statistical analysis of ANP and β-MHC proteins of NRCMs which transfected with AdGFP or AdUSP38 and treated with PBS or Ang II (n=3). Data was calculated by one-way analysis of variance (Tukey's multiple comparisons test). *P < 0.05, **P < 0.01.

To explore the effect of USP38 in cardiomyocyte enlargement, we constructed an effective adenovirus short hairpin RNA-targeting USP38 (AdshUSP38) and adenovirus expressing USP38 (AdUSP38) to transduce NRCMs. The levels of endogenous USP38 expression were significantly downregulated in NRCMs transduced with AdshUSP38, whereas upregulated in NRCMs transduced with AdUSP38 **(Figure S1A and B)**. As shown in **Figure 2A and 2B,** we used immunofluorescent staining to evaluate the cell surface area of NRCMs. The NRCMs were transduced with AdshUSP38 or AdUSP38 for 24 hours, and then treated with Ang II or PBS for 48 hours. Ang II remarkably increased the surface area of NRCMs. Notably, knockdown of USP38 markedly reduced Ang II-caused the increase of cell surface area, whereas USP38 overexpression aggravated the hypertrophied effect. Similarly, the protein levels of ANP and β-MHC were decreased in USP38^cko^ AB mice, but increased in USP38-TG AB mice **(Figure 2C and 2D)**. These results suggest that USP38 serves as a pro-hypertrophic role in Ang II-induced hypertrophied cardiomyocytes.

### Cardiac specific USP38 deficiency alleviates AB-induced pathological cardiac remodeling in mice

**Figure 3:**
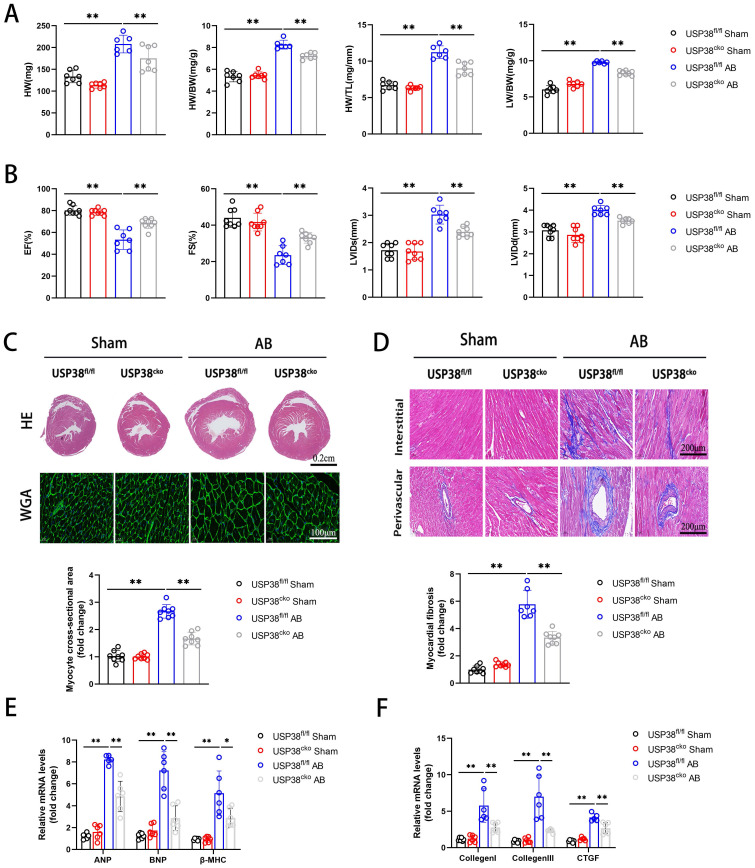
** USP38 deficiency ameliorates AB-induced cardiac remodeling and dysfunction in vivo.** USP38^fl/fl^ and USP38^cko^ mice were subjected to sham or AB for 4 weeks. (A) Heart weight (HW), HW/body weight (BW), Lung weight (LW)/BW and HW/tibia length (TL) ratios from each group (n=6-8). (B) Assessments of echocardiographic parameters of ejection fraction (EF), fraction shortening (FS), left ventricular internal diameter at end-systole (LVIDs), and left ventricular internal diameter at end-diastole (LVIDd) from each group (n=6-8). (C) Representative images of hematoxylin-eosin (H&E) staining (upper), and wheat germ agglutinin (WGA) staining (middle) of LV cross-sections from each group (n=6-8). Scale bar, 200 μm. Quantitative results of average cross-sectional areas (lower) from the indicated groups. (D) Representative images of Masson's staining (upper) of LV cross-sections from each group (n=7-8). Scale bar, 200 μm. (E) The mRNA expression of ANP, BNP, and β-MHC in the heart tissues from each group (n=5-6). Quantitative results of LV interstitial collagen volume (lower) from the indicated groups. (F) The mRNA expression of Collagen I, Collagen III, and CTGF in the heart tissues from each group (n=5-6). Data was calculated by one-way analysis of variance (Tukey's multiple comparisons test). *P < 0.05, **P < 0.01.

To further investigate the function of USP38 in pathological cardiac remodeling, we generated a conditional USP38 deficiency mouse line and the loss of USP38 protein in heart tissues was confirmed in USP38^cko^ mice **(Figure S2)**. USP38^cko^ and USP38^fl/fl^ mice were challenged with sham or AB surgery. As predicted, the HW, HW/BW, HW/TL and LW/BW ratio were significantly increased after AB surgery. However, the harmful effect was alleviated in USP38^cko^ mice **(Figure 3A)**. Furthermore, we found that knockout of USP38 markedly improved pressure overload-cardiac dysfunction, as evidenced by an increase in EF and FS, and a reduction in LVIDs and LVIDd **(Figure 3B)**. In addition, hematoxylin and eosin (H&E) staining and wheat germ agglutinin (WGA) staining unveiled pressure overload-induced pathological cardiac hypertrophy, as demonstrated by an increase in heart size and cardiomyocyte cross-sectional area. Also, the level of myocardial fibrosis was obvious in USP38^fl/fl^ AB mice. Above all hypertrophic and fibrotic phenotypes were dramatically ameliorated in USP38^cko^ mice **(Figure 3C and 3D)**. Consistently, the levels of cardiac hypertrophy related genes (ANP, BNP and β-MHC) and fibrosis related genes (Collagen I, Collagen III and CTGF) were significantly elevated in USP38^fl/fl^ AB mice, while those markers were remarkably decreased in USP38^cko^ mice **(Figure 3E and 3F)**. These results suggest that USP38 deficiency alleviates cardiac hypertrophy and myocardial fibrosis, and prevents the progression of heart failure.

### Cardiac specific USP38 overexpression promotes AB-induced cardiac remodeling in mice

**Figure 4:**
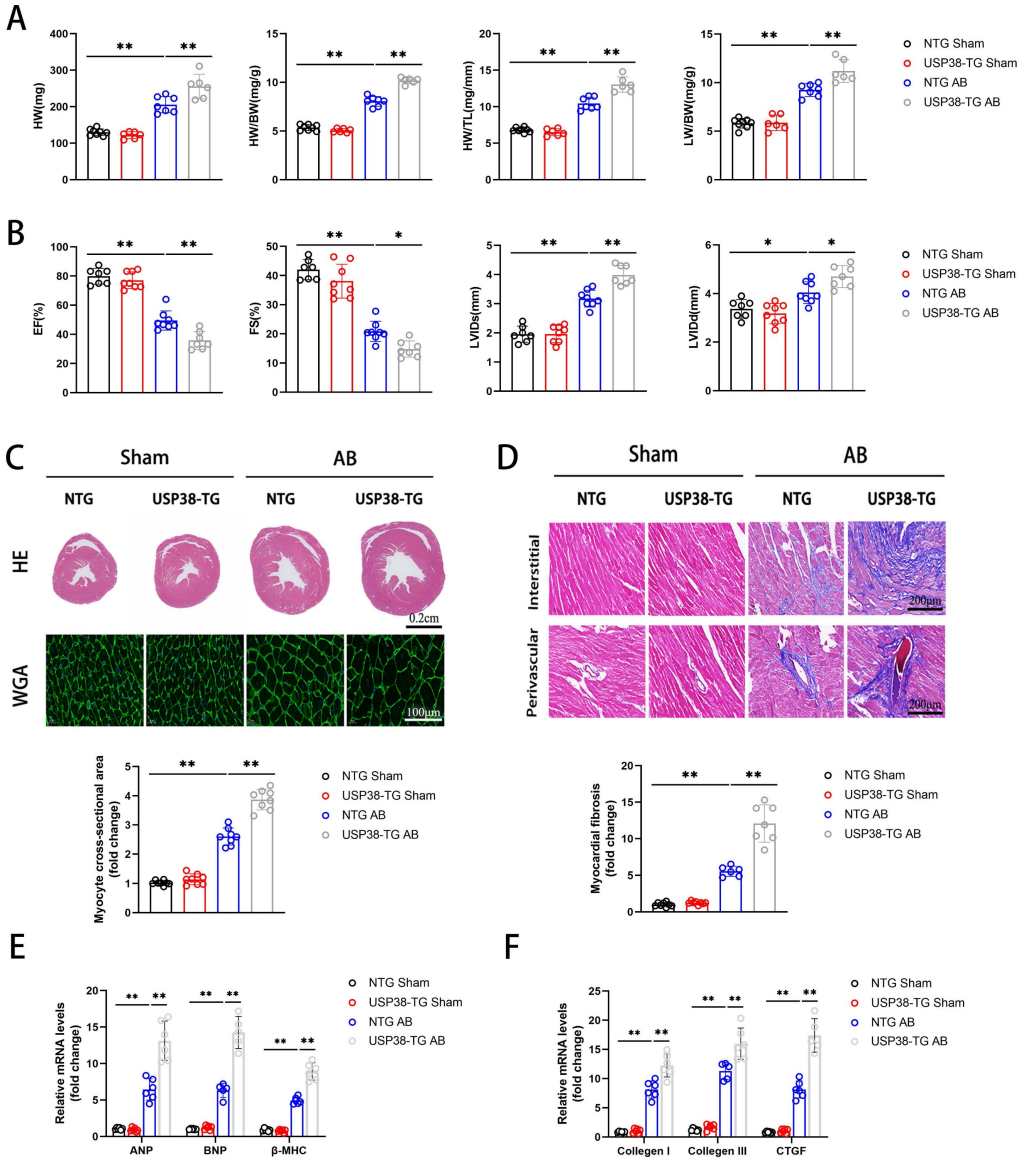
** USP38 overexpression aggravates AB-induced cardiac remodeling and dysfunction in vivo.** NTG and USP38-TG mice were subjected to sham or AB for 4 weeks. (A) The ratio of HW, HW/BW, LW/BW and HW/ TL from each group (n=6-8). (B) Assessments of echocardiographic parameters of EF, FS, LVIDs and LVIDd from each group (n=6-8). (C) Representative images of H&E staining (upper) and WGA staining (middle) of LV cross-sections from each group (n=6-8). Scale bar, 200 μm. Quantitative results of average cross-sectional areas (lower) from the indicated groups. (D) Representative images of Masson's staining (upper) of LV cross-sections from each group (n=6-8). Scale bar, 200 μm. Quantitative results of LV interstitial collagen volume (lower) from the indicated groups. (E) The mRNA expression of ANP, BNP, and β-MHC in the heart tissues from each group (n=5-6). (F) The mRNA expression of Collagen I, Collagen III, and CTGF in the heart tissues from each group (n=5-6). Data was calculated by one-way analysis of variance (Tukey's multiple comparisons test). *P < 0.05, **P < 0.01.

We further evaluated the role of USP38 overexpression in cardiac remodeling by generating cardiac specific USP38 transgenic mouse line (USP38-TG). USP38 overexpression in heart tissues from USP38-TG mice was detected by western blot **(Figure S3)**. We performed AB surgery on USP38-TG and NTG mice, and found the HW, HW/BW, HW/TL and LW/BW ratio of USP38-TG were higher than NTG mice **(Figure 4A)**.

After 4 weeks of AB surgery, echocardiography revealed that EF and FS were decreased and LVIDs and LVIDd were increased in USP38-TG mice compared with NTG mice **(Figure 4B)**. Moreover, USP38-TG mice also displayed greater heart size, cardiomyocyte cross-sectional area and myocardial fibrosis than NTG mice under pressure overload stimulation **(Figure 4C and 4D)**. Consistent to the histological analysis, the mRNA levels of hypertrophic and fibrotic markers were obviously increased in USP38-TG mice **(Figure 4E and 4F)**. Overall, USP38 overexpression aggravates pressure overload-induced pathological cardiac remodeling and promotes the development of heart failure.

### USP38 regulates pathological cardiac remodeling by activating Akt signaling pathway

**Figure 5:**
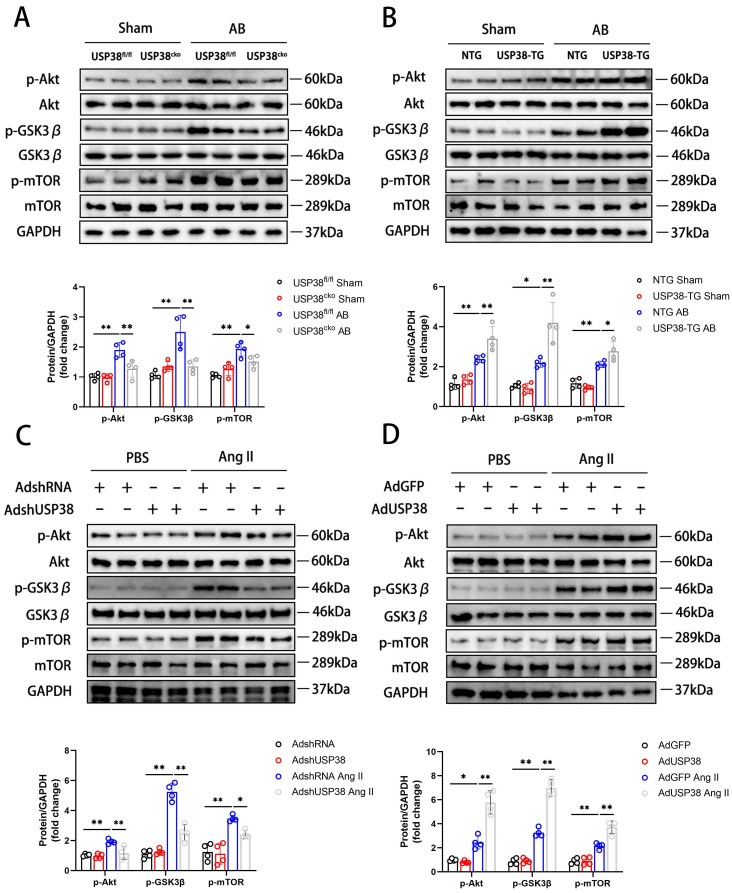
** USP38 activates the Akt signaling pathway in hypertrophic hearts and cardiomyocytes.** (A) Western blot bands and statistical analysis of Akt, p-Akt, GSK3β, p-GSK3β, mTOR and p-mTOR proteins in the hearts of USP38^fl/fl^ and USP38^cko^ mice after sham or AB surgery (n=4). (B) Western blot bands and statistical analysis of Akt, p-Akt, GSK3β, p-GSK3β, mTOR and p-mTOR proteins in the hearts of NTG and USP38-TG mice after sham or AB surgery (n=4). (C) Western blot bands and statistical analysis of Akt, p-Akt, GSK3β, p-GSK3β, mTOR and p-mTOR proteins of NRCMs which transfected with AdshRNA or AdshUSP38 and treated with PBS or Ang II (n=4). (D) Western blot bands and statistical analysis of Akt, p-Akt, GSK3β, p-GSK3β, mTOR and p-mTOR proteins of NRCMs which transfected with AdGFP or AdUSP38 and treated with PBS or Ang II (n=4). Data was calculated by one-way analysis of variance (Tukey's multiple comparisons test). *P < 0.05, **P < 0.01.

Combined with the above research results, we investigated the molecular mechanisms by which USP38 positively regulated pathological cardiac remodeling. In pressure overload-induced mouse hypertrophic model, phosphorylation of Akt at Ser473 level and its downstream signals, including p-GSK3β at Ser9 and p-mTOR at Ser2448 showed a robust augmentation, and USP38 deficiency significantly reduced phosphorylation levels of those proteins **(Figure 5A)**. Conversely, Akt, GSK3β and mTOR phosphorylation levels were markedly increased in USP38 overexpression mice **(Figure 5B)**. Consistently, knockdown of USP38 in NRCMs remarkedly decreased Akt, GSK3β and mTOR phosphorylation levels after Ang II stimulation **(Figure 5C)**, whereas the activation of Akt signaling was obviously enhanced through USP38 overexpression **(Figure 5D)**. In addition, the decreased phosphorylation levels of Akt, GSK3β and mTOR were found in deubiquitinase inactive mutant of USP38 (H857A) under Ang II stimulation **(Supplementary Figure 4A)**. Thus, these data suggest that USP38 promotes the Akt-GSK3β/mTOR pathway in pathological cardiac remodeling.

### USP38 interacts with TBK1 and removes K48-linked polyubiquitination chain

**Figure 6:**
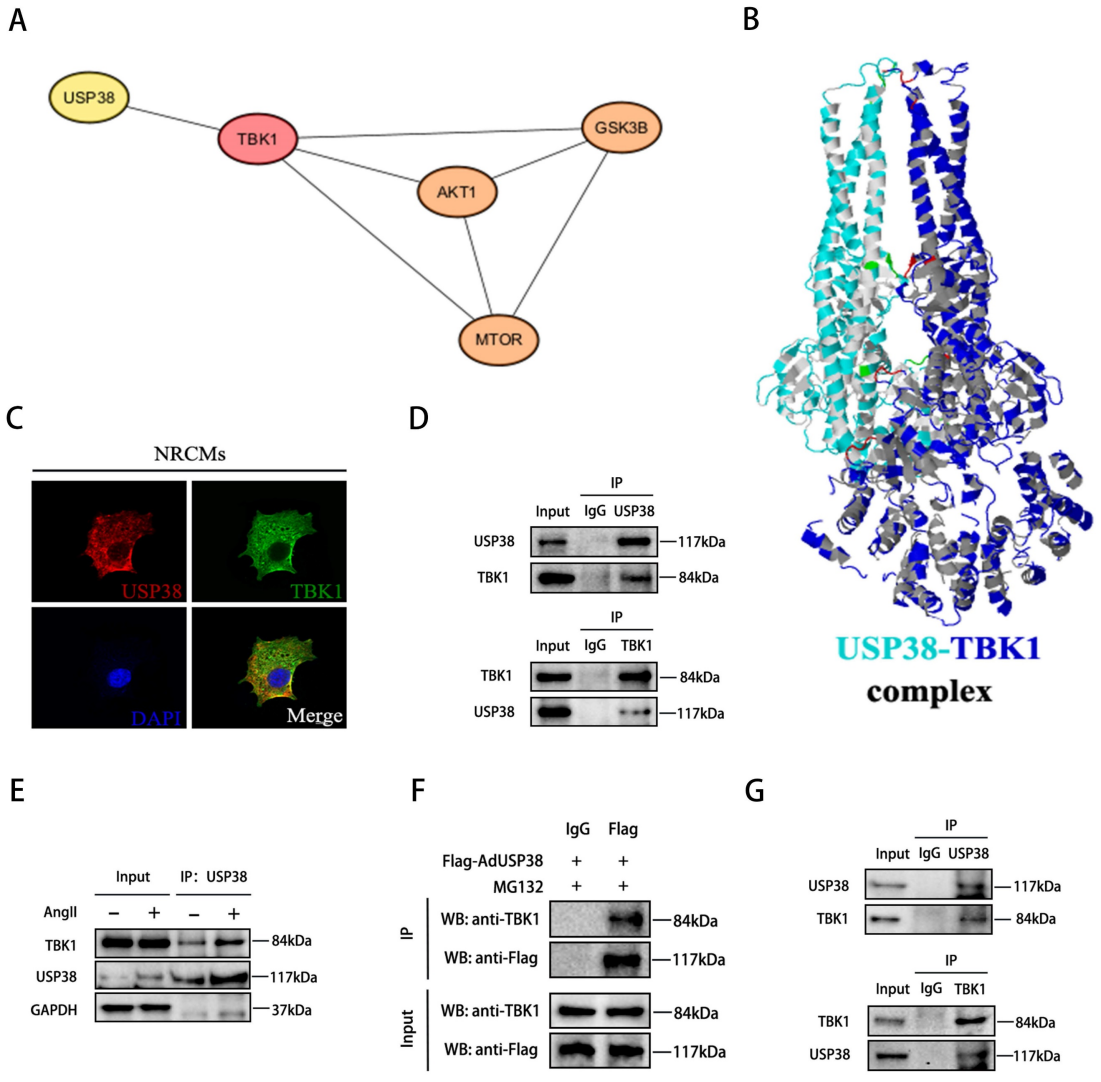
** USP38 interacts with TBK1 in vitro and in vivo.** (A) STRING database identifies protein-protein interaction of USP38, TBK1, Akt, GSK3β and mTOR. (B) Structure-based interacting surface of USP38 and TBK1 model by Z-DOCK. (C) Representative confocal images of the colocalization of USP38 and TBK1 in NRCMs. Scale bar: 20 μm. (D) Endogenous immunoprecipitation analysis of the interaction between USP38 and TBK1 in NRCMs using anti-IgG, anti-USP38 or anti-TBK1. (E) Lysates from NRCMs stimulated with Ang II. Equal amounts of protein lysates were immunoprecipitated with anti-USP38 antibody and analyzed by western blotting with the indicated antibodies. (F) After NRCMs were transfected with Flag-AdUSP38 and IgG antibody was used as a negative control, MG132 was added 6 h before harvest. Immunoprecipitation demonstrated the interaction between exogenous USP38 and endogenous TBK1. (G) Endogenous immunoprecipitation analysis of the interaction between USP38 and TBK1 in myocardial tissues using anti-IgG, anti-USP38 or anti-TBK1.

As TBK1 is an upstream component of the Akt signaling pathway, and the TBK1-Akt signaling cascade is known to be significant in pathological cardiac remodeling^4^, we conducted a comprehensive analysis to explore the association between USP38 and TBK1. Utilizing bioinformatic tools, including the STRING database and the Cytoscape platform, we unveiled that USP38 interacts with TBK1 and regulates Akt-GSK3β/mTOR signaling via TBK1 **(Figure 6A)**. To further validate this interaction, we used Z-DOCK to predict the binding domains between USP38 and TBK1, which are predicted to have interaction energies of -5.9 kcal/mol **(Figure 6B)**. On this basis, we examined the co-localization of USP38 with TBK1 in the cytoplasm of NRCMs by utilizing confocal approach **(Figure 6C)**. We subsequently performed Co-IP and identified that USP38 antibody efficiently precipitated TBK1 in NRCMs, and reverse Co-IP further confirmed that TBK1 interacted with USP38 **(Figure 6D)**. Moreover, the interaction of USP38 with TBK1 was obviously enhanced following Ang II administration **(Figure 6E)**. The interaction was furtherly confirmed with NRCMs transfected with Flag-AdUSP38, Co-IP analysis showed exogenous expression of USP38 interacted with endogenous expression of TBK1 **(Figure 6F)**. Consistent with our in vitro findings, the interaction between USP38 and TBK1 was observed in the heart tissue collected from wild-type mice **(Figure 6G)**. These results suggest that USP38 interacts with TBK1 in NRCMs and myocardial tissues.

**Figure 7:**
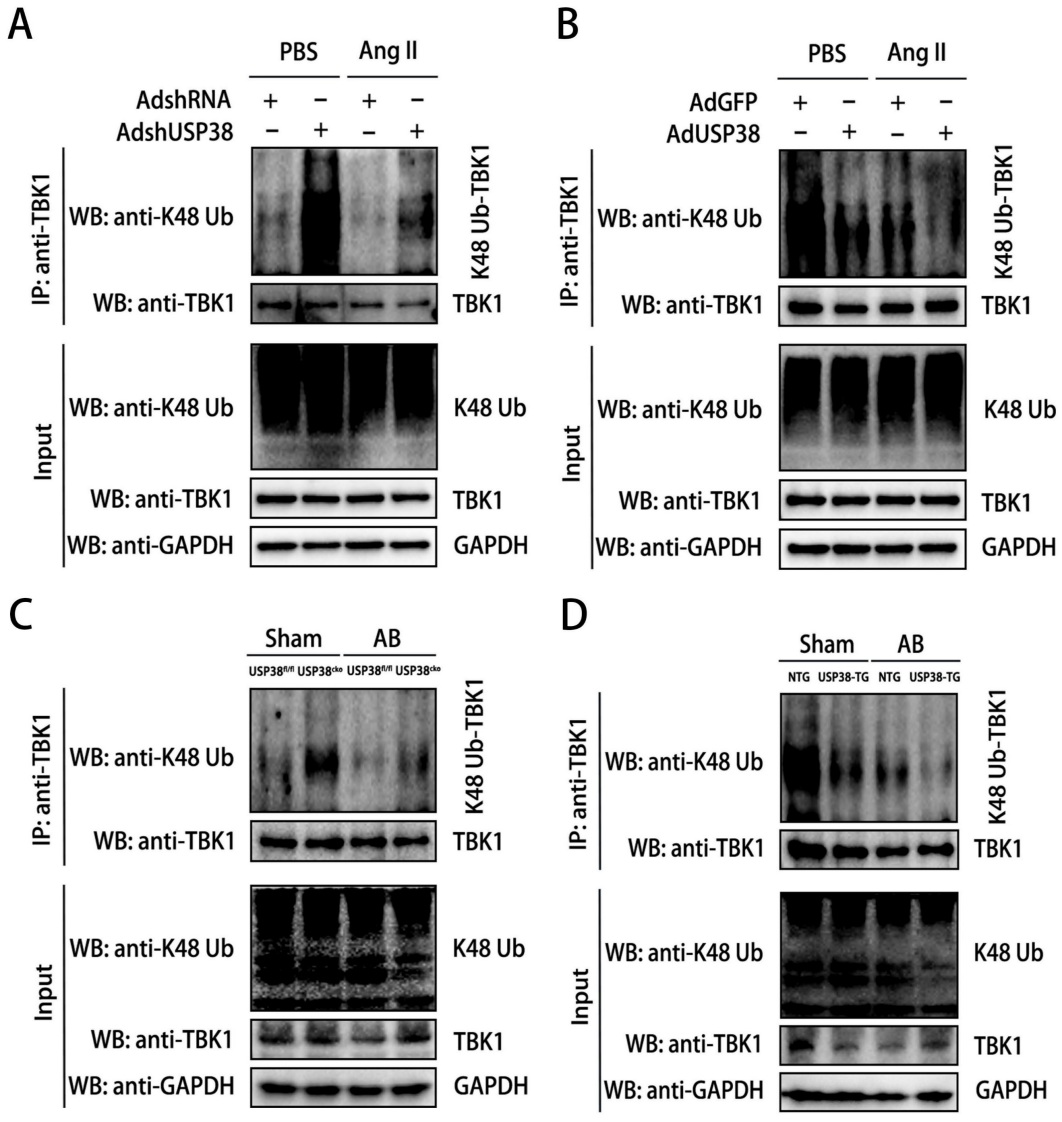
** USP38 removes the K48-linked polyubiquitination level of TBK1.** (A) Result of K48-linked polyubiquitination assays confirming the expression and ubiquitination of TBK1 in NRCMs which transfected with AdshRNA or AdshUSP38 and treated with PBS or Ang II followed by treatment with MG132 for 6 h before harvest. (B) Result of K48-linked polyubiquitination assays confirming the expression and ubiquitination of TBK1 in NRCMs which transfected with AdGFP or AdUSP38 and treated with PBS or Ang II followed by treatment with MG132 for 6 h before harvest. (C) Result of K48-linked polyubiquitination assays confirming the expression and ubiquitination of TBK1 in heart tissue from USP38^fl/fl^ and USP38^cko^ mice after sham or AB surgery. (D) Result of K48-linked polyubiquitination assays confirming the expression and ubiquitination of TBK1 in heart tissue from NTG and USP38-TG mice after sham or AB surgery.

Considering USP38 is a deubiquitinating enzyme, we next explored the role of USP38 on K48-linked polyubiquitination status of TBK1. We firstly examined the K48-linked polyubiquitination level of TBK1 in cellular experiments. After Ang II administration, the K48-linked polyubiquitination level of TBK1 was higher in NRCMs transduced with AdshUSP38 than transduced with AdshRNA **(Figure 7A)**. Conversely, the K48-linked polyubiquitination level of TBK1 was obviously decreased after AdUSP38 transduced NRCMs **(Figure 7B)**, These findings strongly indicate that USP38 interacts with and deubiquitinates TBK1 in hypertrophied cardiomyocytes. Consistent results were also observed in mouse heart tissues** (Figure 7C and 7D)**.

### USP38 prevents the proteasomal degradation of p-TBK1

**Figure 8:**
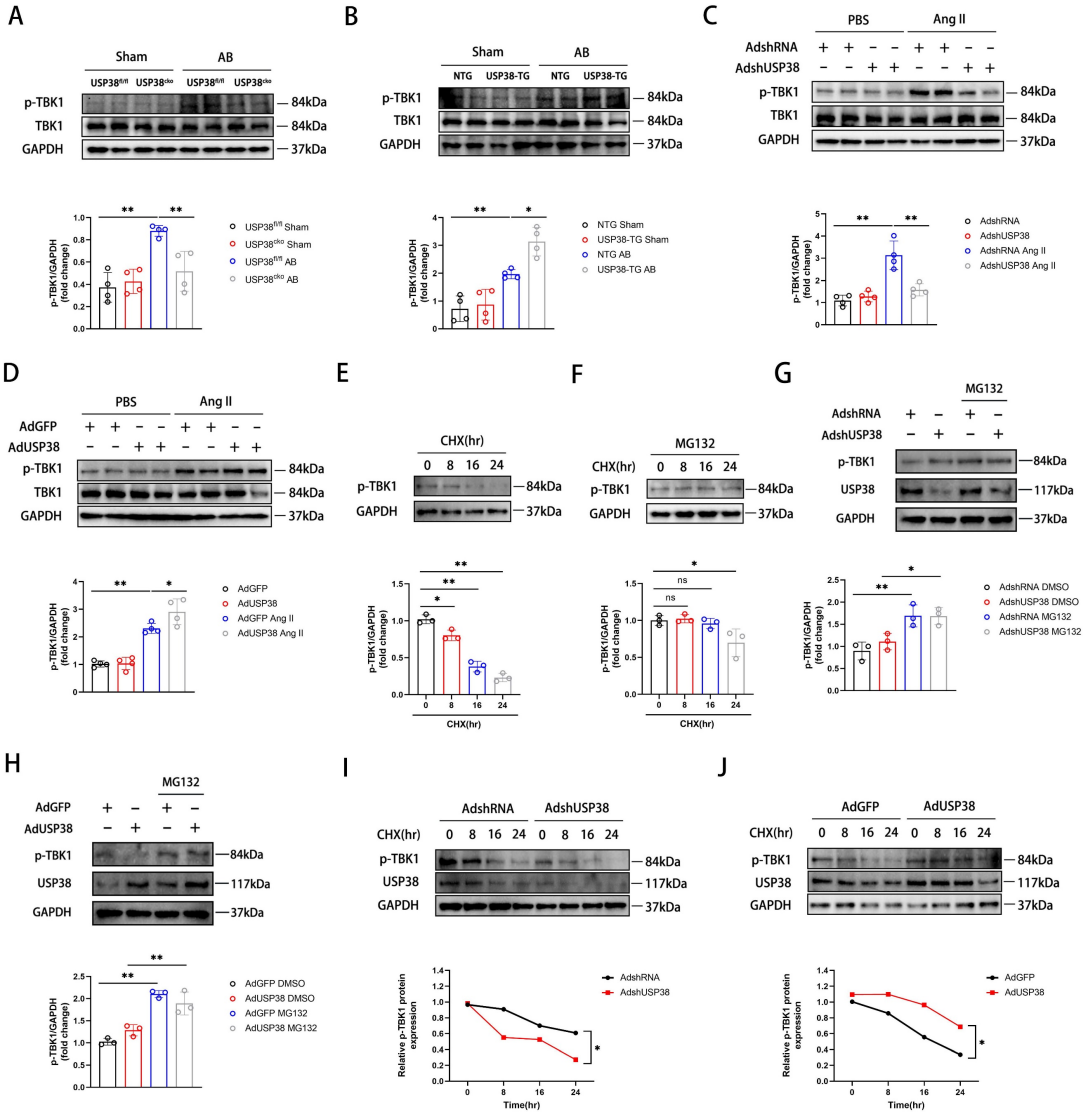
** The degradation of p-TBK1 dependent on the ubiquitin-proteasome pathway.** (A) Western blot bands and statistical analysis of TBK1 and p-TBK1 proteins in the hearts of USP38^fl/fl^ and USP38^cko^ mice after sham or AB surgery (n=4). (B) Western blot bands and statistical analysis of TBK1 and p-TBK1 proteins in the hearts of NTG and USP38-TG mice after sham or AB surgery (n=4). (C) Western blot bands and statistical analysis of TBK1 and p-TBK1 proteins of NRCMs which transfected with AdshRNA or AdshUSP38 and treated with PBS or Ang II (n=4). (D) Western blot bands and statistical analysis of TBK1 and p-TBK1 proteins of NRCMs which transfected with AdGFP or AdUSP38 and treated with PBS or Ang II (n=4). (E) Western blot bands and statistical analysis of TBK1 in NRCMs treated with cycloheximide (CHX, 10 μM) for the indicated times (n=3). (F) Western blot bands and statistical analysis of TBK1 in NRCMs treated with CHX and MG132 for the indicated times (n=3). (G) Western blot bands and statistical analysis of TBK1 in NRCMs which transfected AdshRNA or AdshUSP38 and treated with MG132 or DMSO (n=3). (H) Western blot bands and statistical analysis of TBK1 in NRCMs which transfected AdGFP or AdUSP38 and treated with MG132 or DMSO (n=3). (I) Western blot bands and statistical analysis of TBK1 in NRCMs which transfected with AdshRNA or AdshUSP38 and treated with or without CHX (n=3). (J) Western blot bands and statistical analysis of TBK1 in NRCMs which transfected with AdGFP or AdUSP38 and treated with or without CHX (n=3). Data was calculated by one-way analysis of variance (Tukey's multiple comparisons test) or Student's *t*-test (unpaired, two-tailed, two groups). *P < 0.05, **P < 0.01.

Next, we investigated the impact of USP38 deubiquitinating TBK1. As shown in **Figure 8A and 8B**, USP38 deficiency significantly decreased p-TBK1 level after AB surgery, whereas USP38 overexpression dramatically increased the level of p-TBK1. Consistent with in vivo observations, USP38 knockdown mitigated the Ang II-caused increase of p-TBK1 level, whereas overexpression of USP38 further increased the level of p-TBK1 **(Figure 8C and 8D)**. It is imperative to note that the level of total TBK1 remains unchanged in vivo and in vitro experiment. Building upon these findings, we assessed p-TBK1 levels in NRCMs treated with cycloheximide (CHX) in the absence or presence of the MG132. As expected, the endogenous p-TBK1 protein was decreased by CHX in a time-dependent manner **(Figure 8E)**, which was reversed by MG132 **(Figure 8F)**. Regardless of whether USP38 expression was increased or decreased, the degradation rate of p-TBK1 in NRCMs was notably decreased after MG132 treatment **(Figure 8G and 8H)**. Meanwhile, we found that knockdown of USP38 accelerated the degradation of p-TBK1 induced by CHX, while USP38 overexpression delayed its degradation **(Figure 8I and 8J)**. The results indicate that USP38 may promote the proteasomal degradation of p-TBK1 instead of the total TBK1.

### TBK1 mediated USP38-regulated pathological cardiac remodeling

**Figure 9:**
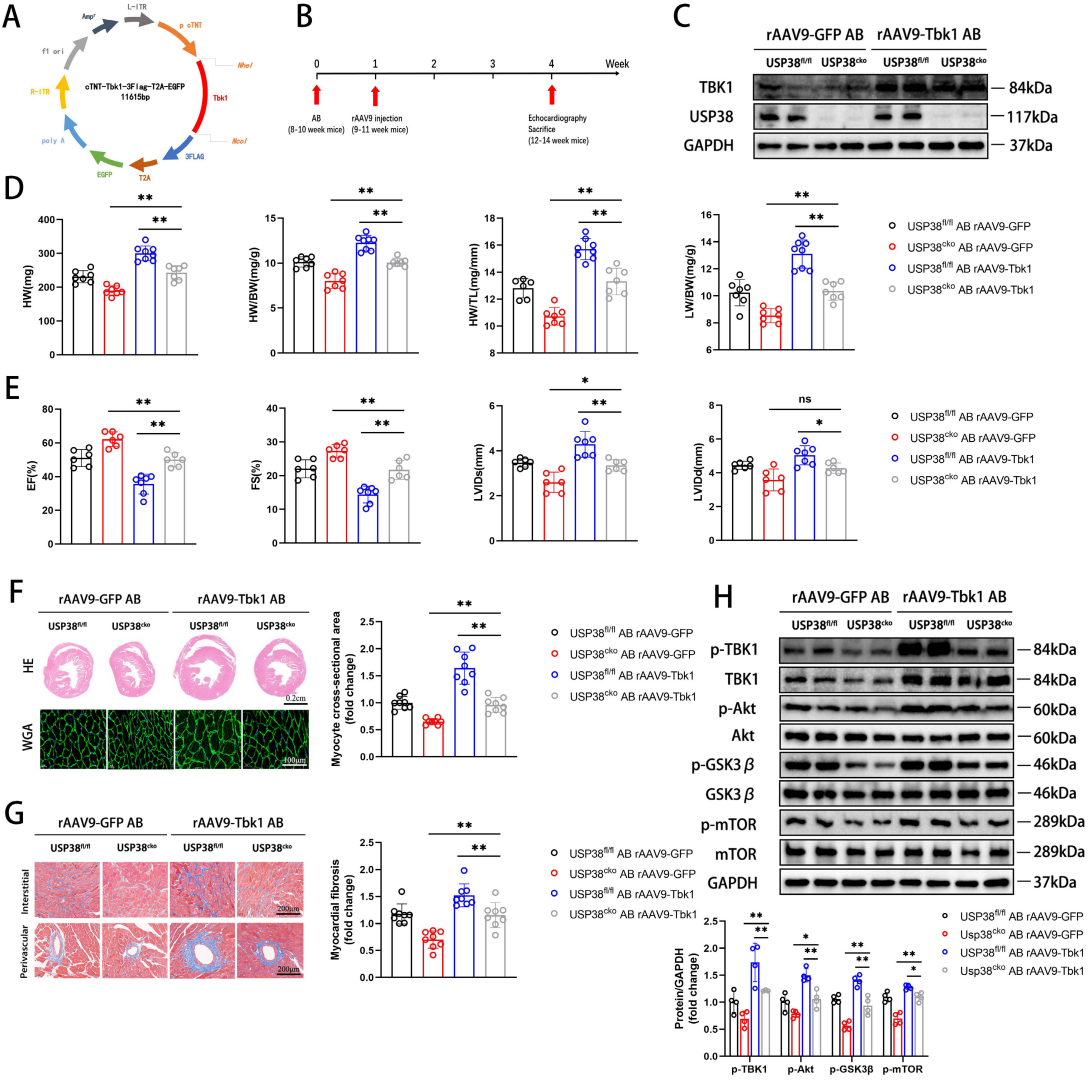
** TBK1 overexpression accelerates cardiac remodeling in USP38^cko^ mice.** USP38^fl/fl^ and USP38^cko^ mice were subjected to sham or AB for 4 weeks, and then injected rAAV9-GFP or rAAV9-Tbk1 at 1 week after surgery. (A) The plasmid for recombinant AAV9 vector overexpressing GFP or TBK1. (B) Protocol for injection of rAAV9 in mouse model of cardiac hypertrophy and remodeling. After being subjected to sham or AB for 1 week, USP38^fl/fl^ and USP38^cko^ mice were injected with rAAV9-GFP or rAAV9-Tbk1. (C) Representative immunoblotting of TBK1 protein in the heart from rAAV9-GFP or rAAV9-Tbk1 mice. (D) The ratio of HW, HWBW, LW/BW and HW/TL from each group (n=7-8). (E) Assessments of echocardiographic parameters of EF, FS, LVIDs and LVIDd from each group (n=6-8). (F) Representative images of H&E staining (upper) and WGA staining (lower) of LV cross-sections from each group (n=6-8). Scale bar, 200 μm. Quantitative results of average cross-sectional areas (right) from the indicated groups. (G) Representative images of Masson's staining (left) of LV cross-sections from each group (n=8). Scale bar, 200 μm. Quantitative results of LV interstitial collagen volume (right) from the indicated groups. (H) Western blot bands and statistical analysis of p-TBK1, p-Akt, p-GSK3β and p-mTOR proteins from each group (n=4). Data was calculated by one-way analysis of variance (Tukey's multiple comparisons test). *P < 0.05, **P < 0.01.

To further confirm the role of USP38-TBK1 axis in pathological cardiac remodeling, we utilized recombinant adeno-associated virus (rAAV9) serotype to overexpress TBK1 **(Figure 9A)**. USP38^cko^ and USP38^fl/fl^ mice were subjected to transduce with rAAV9 one week after AB surgery **(Figure 9B)**. The overexpression of TBK1 in mouse heart was determined by western blot assay **(Figure 9C)**. Compared with the alleviated phenotypes in the pressure overload-induced USP38^cko^, TBK1 overexpression in the heart reduced the protective effect of USP38^cko^ in AB-induced cardiac remodeling, along with increased HW, HW/BW, HW/TL and LW/BW ratio **(Figure 9D)**, exaggerated cardiac dysfunction **(Figure 9E)**, larger myocyte cross-sectional area **(Figure 9F)**, aggravated myocardial fibrosis **(Figure 9G)**, and increased TBK1/Akt signaling protein levels **(Figure 9H)**. These results suggest that TBK1 hyperactivation counteracts the beneficial of USP38 deletion in pathological cardiac remodeling.

**Figure 10:**
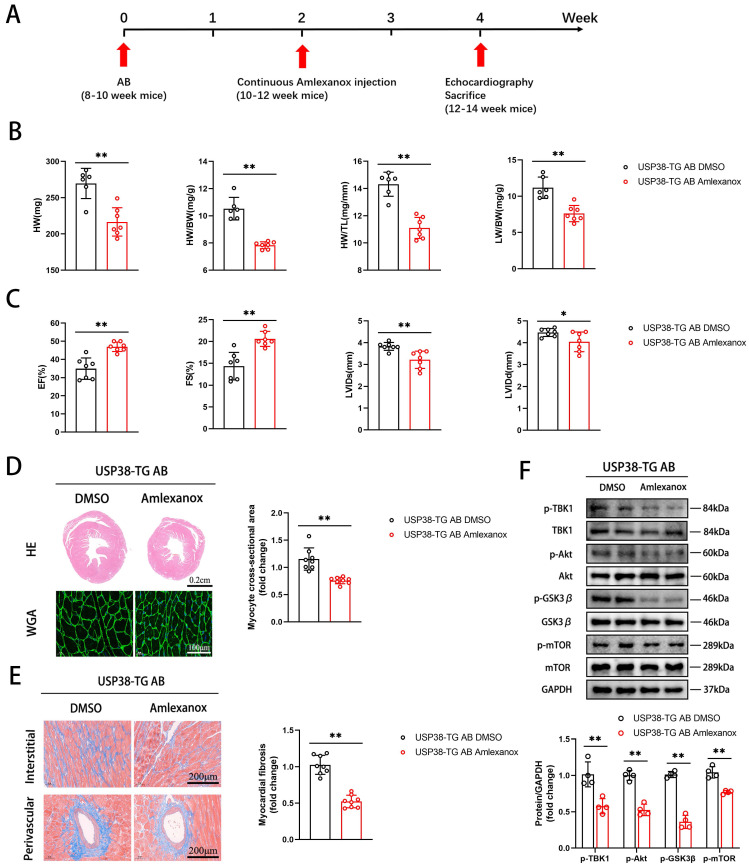
** TBK1 inhibition improves cardiac remodeling in USP38-TG mice.** USP38-TG mice were subjected to AB for 4 weeks, and sequential injected with DMSO or Amlexanox beginning at 2 weeks after surgery. (A) Protocol for injecting DMSO or Amlexanox in mouse model of cardiac hypertrophy and remodeling. After being subjected to sham or AB for 2 weeks, USP38-TG mice were injected with DMSO or Amlexanox for 2 weeks. (B) The ratio of HW, HW/BW, LW/BW and HW/TL from each group (n=6-7). (C) Assessments of echocardiographic parameters of EF, FS, LVIDs and LVIDd from each group (n=6-7). (D) Representative images of H&E staining (upper) and WGA staining (lower) of LV cross-sections from each group (n=6-8). Scale bar, 200 μm. Quantitative results of average cross-sectional areas from the indicated groups. (E) Representative images of Masson's staining (left) of LV cross-sections from each group (n=8). Scale bar, 200 μm. Quantitative results of LV interstitial collagen volume (right) from the indicated groups. (F) Western blot bands and statistical analysis of TBK1, p-TBK1, Akt, p-Akt, GSK3β, p-GSK3β, mTOR and p-mTOR proteins from each group (n=4). Data was calculated by Student's *t*-test (unpaired, two-tailed, two groups). *P < 0.05, **P < 0.01.

Furthermore, we assessed whether inhibition of TBK1 could reverse AB-induced pathological cardiac remodeling. USP38-TG mice were intraperitoneally injected with Amlexanox (an inhibitor of TBK1) or DMSO 2 weeks after AB surgery **(Figure 10A)**. Notably, the detrimental effects in USP38-TG mice were significantly prevented by Amlexanox, as evidenced by reduced HW, HW/BW, HW/TL and LW/BW ratio **(Figure 10B)**, improved cardiac function **(Figure 10C)**, alleviated hypertrophic degree **(Figure 10D)**, ameliorated myocardial fibrosis **(Figure 10E)**, and decreased the protein expression of TBK1/Akt-GSK3β/mTOR signaling pathway **(Figure 10F)** in Amlexanox treated-USP38-TG mice compared with USP38-TG DMSO mice. These results suggest that TBK1 inhibition alleviates the harmful effect of USP38 overexpression on mouse hypertrophic heart. Taken together, we conclude that the exacerbation of USP38-mediated cardiac remodeling is dependent on TBK1.

## Discussion

Herein, we found that USP38 positively regulates pathological cardiac hypertrophy and remodeling by preventing the proteasomal degradation of p-TBK1, leading to the activation of Akt-GSK3β/mTOR signaling pathway and the acceleration of heart failure progression. There findings demonstrate that USP38 is associated with cardiac hypertrophy, which provides a promising therapeutic target for pathological cardiac remodeling.

Previous studies have shown that USPs have potent regulatory effect on cardiac hypertrophy^33^, atherosclerosis^34^, and myocardial ischemia-reperfusion injury^35^, etc. As a crucial member of the USPs family, USP38 has been previously associated with immune diseases and tumors^36^-^38^. USP38 has been reported in regulating many substrates. For example, USP38 has emerged as a novel histone deubiquitinase that involved in modifying KDM5B, HDAC1 and HDAC3, thus regulating inflammation-related genes^37^, genome stability^39^ and cancer stem cell-related genes^38^.

In addition, previous studies have shown the important role of USP38 in cell proliferation by interacting with and deubiquitinating c-Myc^28^, LSD1^27^ and HMX3^40^. Another study demonstrated that USP38 specifically targets Th2 immunity and the associated asthma via removing the K48-linked polyubiquitination of JunB^26^. However, until now, little attention has been paid to the role of USP38 in cardiovascular diseases. In this study, we provided compelling evidence that USP38 is involved in pathological cardiac remodeling. Firstly, we found that the expression of USP38 is increased in heart tissues from patients of heart failure by microarray analysis, and of the top ten DEGs, the mRNA level of USP38 is most significantly upregulated in mouse heart tissues of heart failure. Secondly, we observed a significant increase in USP38 expression by in vivo and in vitro experiments. Finally, our findings unveiled that USP38 deletion remarkably improves pressure overload-induced cardiac dysfunction, cardiac hypertrophy and myocardial fibrosis, accompanied by reduced the transcriptional level of hypertrophic and fibrotic markers, and vice versa.

Recently, a large proportion of USPs have been confirmed to be involved in pathological cardiac remodeling and heart failure. For example, USP4, USP18 and USP19 improves the severity of myocardial hypertrophy and fibrosis by inhibiting transforming growth factor activated kinase 1 (TAK1)^22^, ^23^, ^33^. USP10 is a sirtuin 6 deubiquitinating protein that exerts the protective effect against pathological cardiac hypertrophy^24^. Another notable example is USP25. It has been reported that USP25 deficiency reduces the stability of the SERCA2a protein, which aggravates pressure overload-induced ventricular remodeling^25^. In our study, the mechanism of USP38-mediated cardiac remodeling was explored.

Multiple intracellular signaling pathways contribute to the progression of pathological cardiac remodeling and heart failure^3^, ^41^. Among these, Akt signaling is closely linked to both physiologic and pathological cardiac hypertrophy^42^-^45^. The activation of Akt increases the phosphorylation level of GSK3β and mTOR^4^, ^24^. Previous studies have demonstrated that the reduced hypertrophic responses are shown by inhibiting the GSK3β phosphorylation^46^. The inhibition of mTOR with rapamycin alleviates the degree of cardiac hypertrophy in animal model^47^. During our investigation into the USP38 mediated-signaling pathways, we demonstrated that Akt-GSK3β/mTOR signaling is dramatically activated by USP38 under hypertrophic stimulation. TBK1 has been reported as a positive regulator of cardiac hypertrophy and, importantly, as a potential activator of Akt on cardiac remodeling and heart failure^4^, ^5^, ^48^. Thus, we hypothesized that TBK1 acts as a key node connecting USP38 to Akt signaling in pathologic cardiac remodeling. In the present study, we validated the interaction of USP38 with TBK1 in heart tissue and NRCMs. Recently, ubiquitination modification has been widely studies as a novel mechanism in cardiovascular diseases^49^. K48-linked polyubiquitination serves as a recognition tag for protein degradation dependent on proteasome pathway^50^. It is noteworthy that the degradation of TBK1 plays an important role in antiviral innate immunity by promoting its K48-linked polyubiquitination 16, 17, 51, 52. Additionally, previous studies have demonstrated that USP38 can remove K48-linked polyubiquitination chain^53^. Our study demonstrated that USP38 remarkably decreases K48-linked polyubiquitination level of TBK1 in cardiomyocytes. Furthermore, TBK1 phosphorylation modification is associated with its activation, dephosphorylation of TBK1 usually leads to its inactivation^54^. Hence, the regulation of TBK1 phosphorylation level could be a promising way for pathological cardiac remodeling. Here, we found a reduced phosphorylation level of TBK1 in USP38 deficiency mice after AB surgery, and we demonstrated that the degradation of p-TBK1 is dependent on proteasome pathway.

Finally, the essential role of the USP38-TBK1 axis in pathological cardiac remodeling is demonstrated by “rescue” experiments using rAAV9-Tbk1 (overexpressing TBK1) in USP38^cko^ mice or Amlexanox (TBK1 inhibitor) in USP38-TG mice. We found that TBK1 overactivation partially counteracts the protective effect of USP38 knockout in pathological cardiac remodeling. In contrast, TBK1 inhibitor improves the pro-hypertrophic effects in USP38-TG mice after AB surgery. Therefore, USP38 plays a prominent role in regulating TBK1 activation in hypertrophic myocardium.

## Conclusion

In summary, our present study demonstrated that USP38 positively regulates the progression of pathological cardiac remodeling by stabilizing p-TBK1. We identified a connection between USP38 and TBK1 in hypertrophic myocardium. Thus, interventions targeting the USP38-TBK1 axis may be an effective way to treat pathological cardiac remodeling.

## Supplementary Material

Supplementary figures and tables.
