# Supplementary material for: Ubiquitin specific protease 38 aggravates pathological cardiac remodeling by stabilizing phospho-TBK1
Source: Int J Biol Sci. 2024 Feb 25;20(5):1815–32. doi: 10.7150/ijbs.85562 (PMC10929191; doi:10.7150/ijbs.85562)
Supplement: Supplementary file 1 — Supplementary figures and tables. [file ijbsv20p1815s1.pdf]

1. Supplementary Tables

Table S1 Sequence of primers used for quantitative real-time polymerase chain reaction (qPCR).

Genes	Species	Sequence (5’ → 3’)
Usp5	Mouse	(Forward)-TGTCAGTGTTACCGACGATCC
		(Reverse)-CCGGCGTGTCGAAAGAGAAA
Usp15	Mouse	(Forward)-GTCCCTGCCTCCAGTTCTTGTG
		(Reverse)-GTCCTCCTCCCATCCCTCCATAG
Usp16	Mouse	(Forward)-TTTGGTGGCGAGCTGACTAG
		(Reverse)-CTCCTAGGTCCTCTGGGCTT
Usp22	Mouse	(Forward)-GAGTTCCTCATTGCAGCCCT
		(Reverse)-CTCTTTGCTGGAGGCCATGA
Usp38	Mouse	(Forward)-TCAATCAAAGCGCCTGGACT
		(Reverse)-CCCACAGTTTAGGCAGCAGA
Usp39	Mouse	(Forward)-CATGTACCTGACGCTGGACCTTC
		(Reverse)-CTCCGTGATGCCGTTGAACTTG
Usp42	Mouse	(Forward)-AAGAGTCTGATGAGGAGTCGAA
		(Reverse)-CGCTATTAGCACCATTTAGCAG
Usp51	Mouse	(Forward)-AGTTGATGGCATCTGAGGTGG
		(Reverse)-ATAAGGCCCAGGCAAATCCAA
Usp52	Mouse	(Forward)-TCGTCCACCCTACTCTTCACACTC
		(Reverse)-GCTCCAGGCAGATACTTCGCTTC
Usp53	Mouse	(Forward)-AAGCCTAGCGGCAATCTTGG
		(Reverse)-GTTCTGCCCTGGCTCGTTTA
ANP	Mouse	(Forward)-TCGTCTCCTTTTGGCT
		(Reverse)-TCCAGGTGGTCTAGCAGGTTCT
BNP	Mouse	(Forward)-AAGTCCTCGCCAGTCTCCAGA
		(Reverse)-GAGCTGTCTCTGGGCCATTTC

β-MHC	Mouse	(Forward)-CCGAGTCCCAGGTCAACAA
		(Reverse)-CTTCACGGGCACCCTTGGA
Collengen I	Mouse	(Forward)-AGGCTTCAGTGGTTTGGATG
		(Reverse)-CACCAACAGCACCATCGTTA
Collengen III	Mouse	(Forward)-CCCAACCCAGAGATCCCATT
		(Reverse)-GAAGCACAGGAGCAGGTGTAGA
CTGF	Mouse	(Forward)-AAAGCAGCTGCAAATACCAATG
		(Reverse)-AAATGTGTCTTCCAGTCGGTAG
GAPDH	Mouse	(Forward)-ACTCCACTCACGGCAAATTC
		(Reverse)-TCTCCATGGTGGTGAAGACA

Table S2 Primary antibodies for Western-blots

Primary antibodies	Source organism	Producer	Number
USP38	Rabbit	Proteintech	17767-1-AP
TBK1	Rabbit	CST	#3504
p-TBK1	Rabbit	CST	#5483
Akt	Rabbit	CST	#9272
p-Akt	Rabbit	CST	#4060
GSK3β	Rabbit	CST	#9315
p-GSK3β	Rabbit	CST	#9322
mTOR	Rabbit	CST	#2983
p-mTOR	Rabbit	CST	#2971
ANP	Rabbit	Abcam	Ab225844
β-MHC	Rabbit	Proteintech	22280-1-AP
IgG	Rabbit	CST	8726S
Flag	Rabbit	Abclonal	AE169
K48-linkage polyubiquitin	Rabbit	CST	#4289
GAPDH	Rabbit	CST	#5174S

2. Supplementary Figures

Figure S1

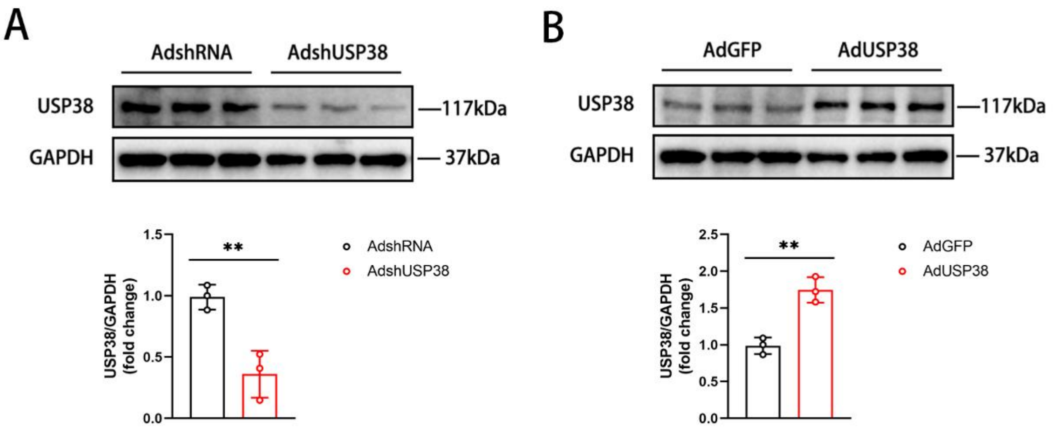

Figure S1. The efficiency of adenovirus in NRCMs. (A) Western blot bands and statistical analysis of USP38 in NRCMs which transfected with AdshRNA or AdshUSP38 for 24 hours (n=3). (B) Western blot bands and statistical analysis of USP38 in NRCMs which transfected with AdGFP or AdUSP38 for 24 hours (n=3). Data was calculated by Student's t-test (unpaired, two-tailed, two groups). *$P < 0.05$, **$P < 0.01$.

Figure S2

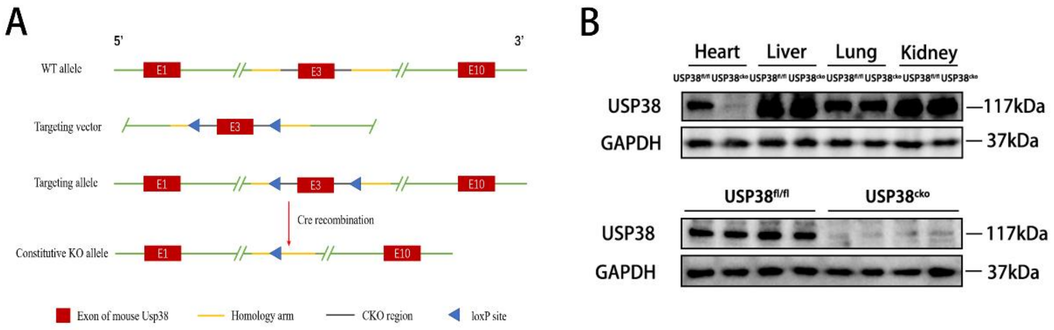

Figure S2. The efficiency of cardiac-specific USP38 knockout in mice. (A) Schematic diagram describing the strategy for the generation of cardiac-specific USP38 knockout mice. (B) Representative immunoblotting of USP38 protein in the heart, liver, lung and kidney from USP38^{cko} and USP38$^{fl/fl}$ mice.

Figure S3

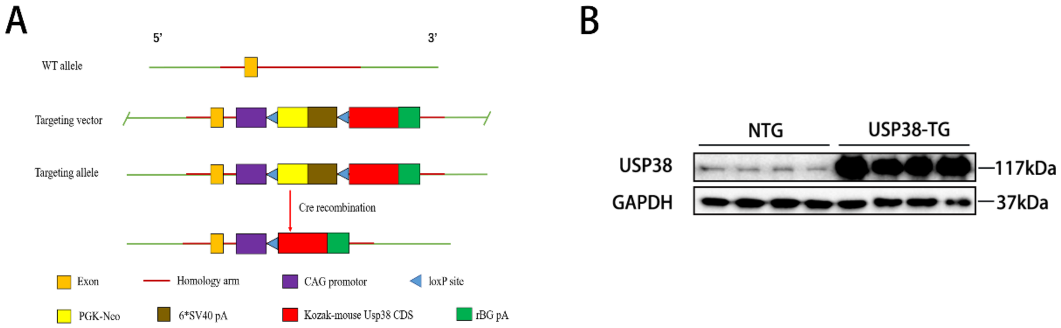

Figure S3. The efficiency of cardiac-specific USP38 overexpression in mice. (A) Schematic diagram describing the strategy for the generation of cardiac-specific USP38 overexpression mice. (B) Representative immunoblotting of USP38 protein in the heart from USP38-TG and NTG mice.

Figure S4

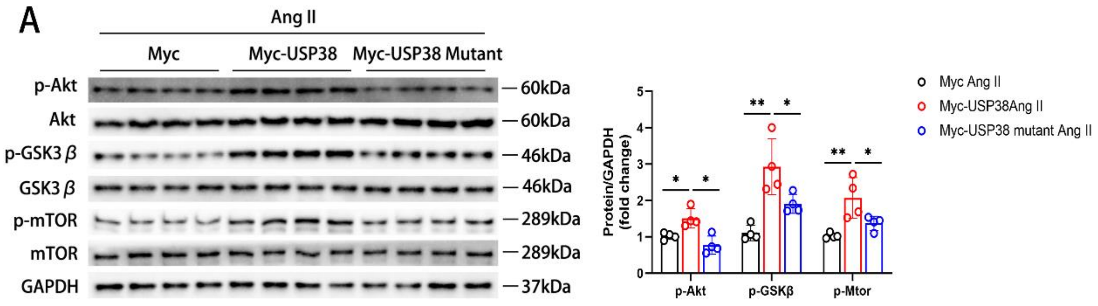

Figure S4. Loss of USP38 activity hamper the activation of Akt signaling pathway. (A) Western blot bands and statistical analysis of Akt, p-Akt, GSK3β, p-GSK3β, mTOR, and p-mTOR proteins (n=4). Data was calculated by one-way analysis of variance (Tukey's multiple comparisons test). $^*P < 0.05$, $^{**}P < 0.01$.
